# Synthesis of High-Purity Hydroxyapatite and Phosphoric Acid Derived from Moroccan Natural Phosphate Rocks by Minimizing Cation Content Using Dissolution–Precipitation Technique

**DOI:** 10.3390/molecules29163854

**Published:** 2024-08-14

**Authors:** Karim Benataya, Mohammed Lakrat, Othmane Hammani, Mohamed Aaddouz, Youssef Ait Yassine, Hatem A. Abuelizz, Abdelkader Zarrouk, Khalid Karrouchi, Elmiloud Mejdoubi

**Affiliations:** 1 Laboratory of Applied Chemistry and Environment, Department of Chemistry, Faculty of Sciences, Mohammed First University, Oujda 60000, Morocco; k.benataya@ump.ac.ma (K.B.); m.lakrat@ump.ac.ma (M.L.); m.aaddouz@ump.ac.ma (M.A.); ee.mejdoubi@gmail.com (E.M.); 2 Chemistry Platform, Unités d’Appui Technique à la Recherche Scientifique (UATRS), Centre National pour la Recherche Scientifique & Technique (CNRST), Rabat 10102, Morocco; othmane.hammani@hotmail.com; 3 Higher School of Technology, Ibn Zohr University, Laayoune 3007, Morocco; y.aityassine@uiz.ac.ma; 4 Laboratory of Thermodynamics and Energy, Faculty of Sciences, Ibn Zohr University, Agadir 80150, Morocco; 5 Department of Pharmaceutical Chemistry College of Pharmacy, King Saud University, P.O. Box 2457, Riyadh 11451, Saudi Arabia; habuelizz@ksu.edu.sa; 6 Laboratory of Materials, Nanotechnology and Environment, Faculty of Sciences, Mohammed V University in Rabat, Rabat P.O. Box 1014, Morocco; azarrouk@gmail.com; 7 Research Centre, Manchester Salt & Catalysis, 88-90 Chorlton Road, Manchester M15 4AN, UK; 8 Laboratory of Analytical Chemistry and Bromatology, Team of Formulation and Quality Control of Health Products, Faculty of Medicine and Pharmacy, Mohammed V University in Rabat, Rabat 10100, Morocco

**Keywords:** hydroxyapatite, natural phosphate, heavy metals, precipitation–dissolution, decontamination

## Abstract

This study investigates, in the first part, the synthesis and purification of a poorly crystalline hydroxyapatite (HAp) using natural Moroccan phosphate (Boucraa region) as a raw material. Despite its successful preparation, the obtained HAp was contaminated by several metallic cations (mostly Cd, Pb, Sn, Ti, Mn, Mg, Fe, and Al) migrated from the natural rocks during the digestion process, inhibiting HAp application in several sectors. To minimize the existence of these elements, the dissolution–precipitation technique (DP) was investigated as a non-selective purification process. Following the initial DP cycle conducted on the precipitated HAp, the removal efficiency was approximately 60% for Al, Fe, Mg, Mn, and Ti and 90% for Cd and Pb. After three consecutive DP cycles, notable improvement in the removal efficiency was observed, reaching 66% for Fe, 69% for Mg, 73% for Mn, and 74% for Al, while Cd, Pb, and Ti were totally removed. In the second part of this study, the purified HAp was digested using sulfuric acid to produce high-quality phosphoric acid (PA) and gypsum (GP). The elemental analysis of the PA indicates a removal efficiency of approximately 89% for Fe and over 94% for all the examined cations. In addition, the generated GP was dominated by SO_3_ and CaO accompanied with minor impurities. Overall, this simple process proves to be practically useful, to reduce a broad spectrum of cationic impurities, and to be flexible to prepare valuable products such hydroxyapatite, phosphoric acid, and gypsum.

## 1. Introduction

Hydroxyapatite (HAp: Ca_10_(PO_4_)_6_(OH)_2_) is a well-known calcium phosphate compound that has witnessed considerable interest in various fields owing to its unique properties. As a major component of the human bone and teeth, HAp is recognized as an attractive biomaterial in regenerative medicine [1], dentistry [2], cancer therapy [3], bioimaging [4], and drug delivery [5]. Beyond its traditional medical applications, researchers explored HAp utilities in other fields including catalysis [6,7], fertilization [8], and adsorption [9] as well as in the cosmetic and pharmaceutical industries [10]. Therefore, the preparation of HAp has seen huge advancements either by improving the already existing protocols such as the sol-gel process [11], hydrothermal [12], microwave irradiation [13], spray pyrolysis [14], or exploring novel techniques involving wastes and mineral resources [15,16]. One of the approaches that is paving the way for HAp preparation is the valorization of natural phosphate as a low-cost and abundant natural source, especially in Morocco [6,16,17]. These studies primarily focus on the digestion of sedimentary rocks to generate a crude liquor (c-Liq) rich in calcium and phosphorous ions, which is then precipitated into HAp using an alkaline solution. However, there is a notable absence of studies focusing on the purification of the precipitated phases. Indeed, sedimentary rocks commonly contain calcium phosphate minerals, particularly apatite (Ca_10_(PO_4_)_6_(OH,F,Cl)_2_), quartz (SiO_2_), and dolomite (Mg Ca (CO_3_)_2_) along with an important amount of heavy metals and rare earth elements [18]. As a result, during the digestion process, these hazardous elements migrate into the c-Liq, which, when it is used in the preparation of HAp, they integrate into its structure as contaminants. Taking into consideration their non-biodegradability, toxicity, as well as carcinogenicity, the presence of these cations in the final product poses significant concerns regarding human health [19,20]. Hence, it becomes crucial to effectively remove or minimize the presence of these hazardous elements prior to HAp synthesis to ensure the purity and quality of the final product. To address these limitations, several techniques have been suggested for purifying the digested c-Liq such as solvent and liquid–liquid extraction [21], crystallization [22], extractant impregnated resin process [23], membrane [24], and photocatalytic technologies [25]. Despite the considerable efforts, decontamination techniques continue to face notable environmental, economic, and technical limitations, such as complex purification processes, limited efficiency, and high costs. Conversely, precipitation methods have been regarded as economic and promising strategies for the c-Liq purification [26,27]. Meanwhile, most of these methods are typically selective, focusing on the removal of a specific cation while permitting other impurities to persist in the c-Liq. They commonly rely on the precipitation of the hazardous elements as insoluble salts. This includes the defluorination through MgSiF_6_·6H_2_O or (Na,K)_2_SiF_6_ precipitation [28]; heavy metal precipitation as sulfides (FeS_2_, CuS, PbS, and CdS) [29], ferrous oxalate (FeC_2_O_4_·2H_2_O) [30], ferric oxide (Fe_2_O_3_) or ferric oxyhydroxide (Fe(OH)_3_) [31]; and magnesium precipitation as MgSiF_6_, MgAlF_5_, or MgAl_2_F_8_ compounds [32].

Therefore, the development of an innovative concept to purify the produced HAp could represent a potential avenue to maximize the valorization of natural phosphate resources. In this study, we explored a promising method to eliminate a broad range of metallic ions from the c-Liq and produce less-contaminated HAp via a wet process utilizing the dissolution–precipitation technique. The suggested method is a non-selective two-step process to clean the prepared HAp from cationic impurities offering an inexpensive and rapid decontamination approach. In the first step, the c-Liq digested from phosphate rocks was transformed into HAp using ammonia solution at room temperature. This ensures the formation of a solid phase containing calcium and phosphate ions with fewer hazardous elements. Next, HAp was dissolved using diluted nitric acid to recover its constituent elements, facilitating the evaluation of their elimination efficiency. To improve the purification process, three consecutive DP cycles were conducted on the same HAp powder.

Following both the first and third cycles, we examined the structure of the obtained HAp and assessed the concentration of residual cations in the recovered liquor (r-Liq).

In the final stage, the purified HAp obtained after the third DP cycle was treated with sulfuric acid to generate high-quality PA and GP. Both the chemical compositions of PA and GP and the structural properties of GP were subsequently analyzed.

## 2. Results and Discussion

**Figure 1:**
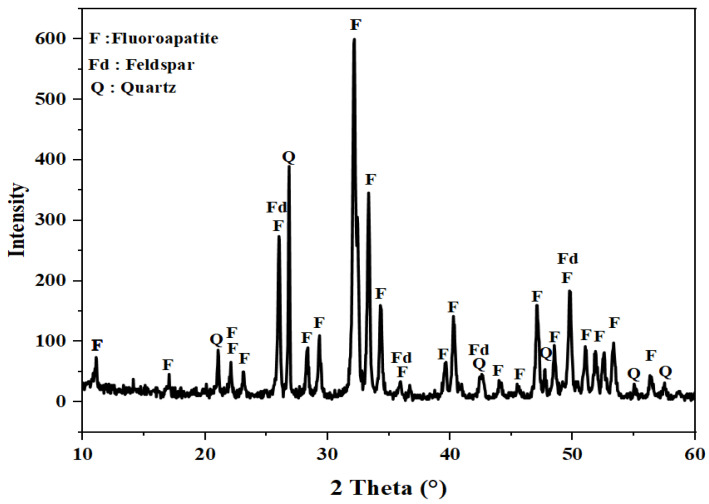
XRD pattern of the natural phosphate calcined at 900 °C.

The XRD pattern of the calcined natural phosphate (Figure 1) revealed that the dominant minerals are fluorapatite, quartz, and feldspar [33].

The mineral composition of the calcined natural phosphate was determined using XRF spectrometry. As given in Table 1, the phosphate rocks are principally composed of P_2_O_5_, CaO, SiO_2_, and F with mass fractions of 39.58%, 44.01%, 5.26%, and 5.12%, respectively. The presence of other cations which are considered as hazardous elements including Al, Mn, Mg, Fe, S, Na, and Ti was also confirmed.

Table 2 presents the chemical composition of the c-Liq derived from phosphate rock digestion. The results indicate a dominance of phosphorus and calcium ions, accompanied by various impurities including aluminum, iron, magnesium, titanium, manganese, and cadmium. It is worth noting that in the following sections, the study is specifically concentrated on the evaluation of cations such as Fe, Al, Mg, Mn, Ti, Cd, Sn, and Pb and their removal efficiency. Although the XRF analysis also detected anions, they are not the primary focus of this work and will not be discussed further.

As it was expected, the heavy metals detected earlier in the phosphate rocks end up in the c-Liq during the digestion process. Therefore, the separation of all these cations from this solution constitutes a major problem to produce a valuable product such as HAp with minimal contamination. The purification process proposed herein is based on the conversion of the c-Liq rich in phosphor and calcium ions into a solid phase, particularly HAp, using ammonia solution. In this sense, many studies tried to remove impurities from the contaminated solution using adsorption and extraction techniques. However, the main idea behind the present work is to extract phosphorus and calcium from impurities by inducing their precipitation as a solid phase that can be easily separated out through a simple filtration process.

**Figure 2:**
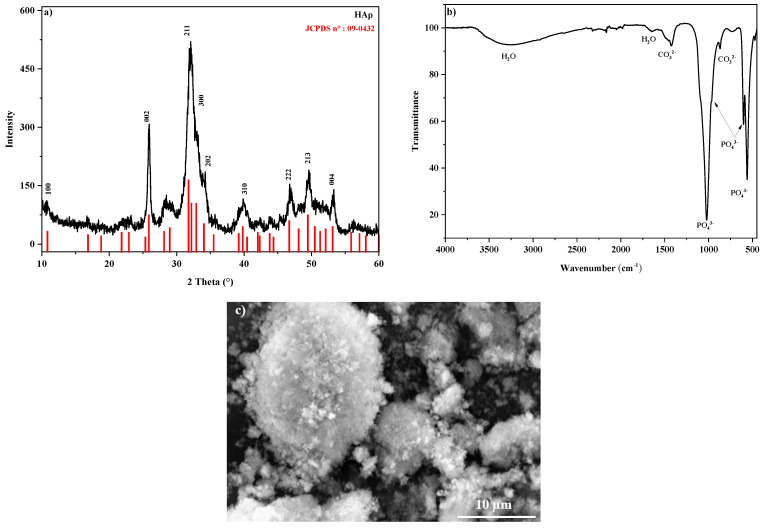
(**a**) XRD pattern; (**b**) ATR−FTIR spectra; and (**c**) SEM image of the prepared HAp.

The XRD pattern (Figure 2a) of the precipitated solid phase displayed diffraction peaks that match well with those of the hexagonal hydroxyapatite structure (JCPDS No. 09-0432) [34]. Moreover, the broad diffraction peaks observed indicate that the prepared HAp has low crystallinity.

The ATR-FTIR spectrum of the obtained sample (Figure 2b) reveals the presence of typical absorption bands of an apatitic structure. The three absorption bands appearing at 463, 560, and 603 cm^−1^ are related to the bending vibration of phosphate groups (PO_4_^3−^), while those observed around 960, 1020, and 1089 cm^−1^ correspond to the stretching vibration of (PO_4_^3−^) groups [34]. The broad bands located between 3300–3450 cm^−1^ and the relatively small band at 1620 cm^−1^ are corresponding to water (H_2_O) molecules adsorbed on the HAp particles’ surface [35]. A few additional bands were detected at 1580, 1415, and 873 cm^−1^, probably due to the presence of carbonate ions CO_3_^2−^ [36].

The morphology of the precipitated phases is given in Figure 2c, indicating the presence of agglomerated particles in the nanometer size range.

**Figure 3:**
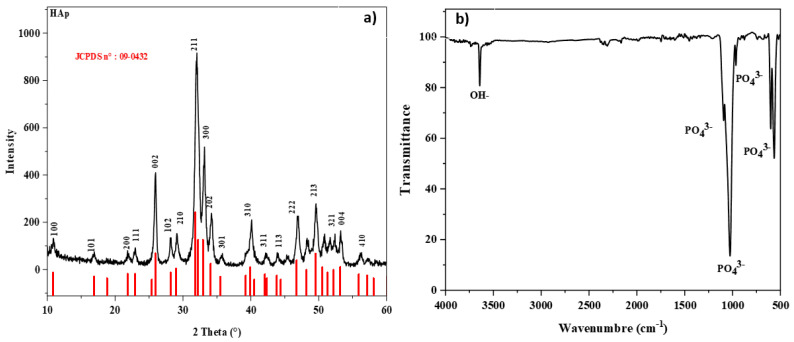
(**a**) XRD pattern and (**b**) ATR−FTIR spectra of precipitated sample after calcination at 900 °C.

The XRD pattern and ATR-FTIR spectrum of the calcined powder (Figure 3a) showed the existence of a well-crystallized phase corresponding to HAp without noticing any other crystalline phase.

To evaluate the effectiveness of the precipitation step in reducing impurities, the produced HAp was totally transformed into a clear solution via a simple dissolution step [37]. Thus, HAp was subjected to reaction with diluted nitric acid, serving as a leaching reagent.

There were two main purposes for using nitric acid instead of sulfuric acid for leaching phosphate rocks to produce the c-Liq. Firstly, to avoid the generation of a large volume of contaminated GP as a byproduct, and secondly, to keep sufficient calcium ions in the c-Liq to be utilized for HAp precipitation. For instance, a complete dissolution of HAp without secondary precipitation occurs, enabling the delivery of its content in the solution, allowing us to precisely determine its chemical composition.

The elemental composition of the r-Liq is highlighted in Table 3 where the results indicate a significant decrease in the impurities content following the precipitation of HAp from the crude liquor.

**Figure 4:**
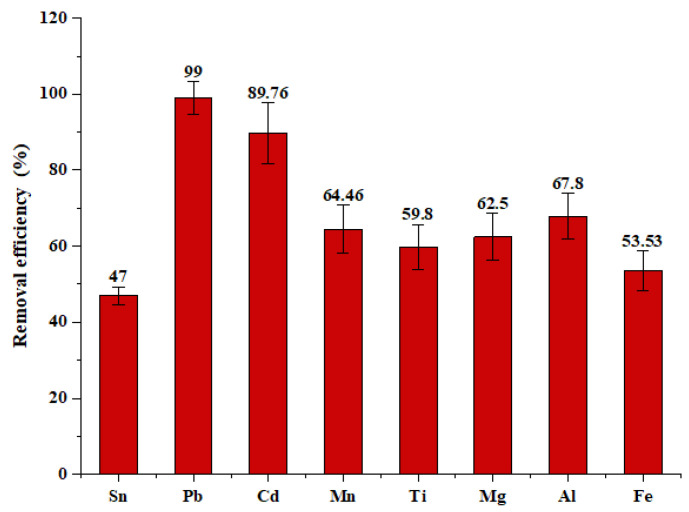
Removal efficiency of different cations.

Analyzing the removal efficiency (Figure 4), it is evident that the precipitation of the phosphorus and calcium ions existing in the c-Liq into HAp effectively eliminated a broad spectrum of metal ions. Specifically, the removal rates were 47.01% for Sn, 89.76% for Cd, and 99.02% for Pb, while Mn and Ti exhibited removal rates close to 60%. For the main elements such as Mg, Fe, and Al, the removal percentage ranged mainly between 53% and 65%.

Mechanisms responsible for this decontamination process can be attributed to the pH change in the c-Liq inducing a fast precipitation of HAp. From a physicochemical point of view, the precipitation of HAp is thermodynamically favorable in the Ca^2+^-PO_4_^3−^-H_2_O system at a pH value higher than 7 more than any other calcium phosphate phase [38]. The presence of the hazardous elements (impurities) in the r-Liq is probably due to their incorporation in the apatite lattice or simply adsorbed on its surface during precipitation. In fact, HAp presents a flexible hexagonal structure that can accommodate almost all the cations within its crystal lattice as substituents for Ca^2+^ ions [39,40]. In various chemical contexts, calcium ions can be substituted by numerous metallic cations including alkali metals (Li^+^, Na^+^, K^+^…), alkaline earth metals (Ba^2+^, Mg^2+^, Sr^2+^…), transition metals (Mn^2+^, Cu^2+^, Ni^2+^, Zn^2+^, Cd^2+^, and Co^2+^) as well as a variety of other cations (Pb^2+^, Al^3+^, and Fe^3+^) [9]. Therefore, rapid filtration of the solid phase was carried out with the aim to minimize the exchange between calcium in the HAp structure and cations in the c-Liq which facilitate the recovery of less-contaminated compounds. What is more, the fast increase in the pH of liquid to around 9 was performed to prevent the formation of secondary solid phases such as iron phosphate (FePO_4_), aluminum phosphate (AlPO_4_), and chromium phosphate (CrPO_4_), which are usually precipitated, respectively, in the pH ranges of 0.3–2.6, 2.0–2.5, and 4–6 [41]. The absence of these phases as confirmed by XRD analysis of the non-sintered (Figure 2) and sintered (Figure 3) powders reflecting the presence of a single phase, which is HAp.

Although the precipitation step significantly decreased impurities from the produced HAp, it did not achieve a complete decontamination.

To address this, a series of DP cycles was proposed as a simple and effective strategy to further reduce the presence of impurities in the prepared HAp derived from natural phosphate. Essentially, during the recrystallization process, crystals tend to expel impurities, allowing the recuperation of less-contaminated compounds. By repeating this process, it may be possible to entirely eliminate the studied cations from the main HAp crystal structure.

Elemental analysis of the recovered liquids r1-Liq and r3-Liq (Table 4) after the first and third DP cycle, respectively, showed a noticeable diminution in the concentration of each hazardous element, highlighting the relationship between the DP cycle and their removal efficiency.

**Figure 5:**
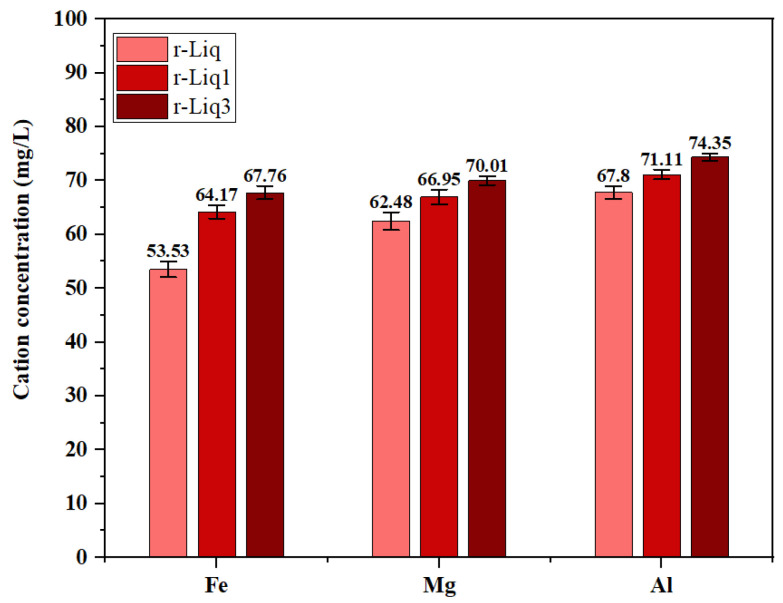
Removal efficiency of Fe, Mg, and Al ions after the first and third DP cycles.

In fact, it is obvious that the concentration of the different cationic species coexisting in the recovered liquor decreased almost linearly with each consecutive DP cycle. The concentrations of Sn and Pb were below the detection limits after the first cycle, indicating their full elimination from the HAp structure. For Cd and Ti, their elimination was complete after three consecutive PD cycles performed on the same sample. Furthermore, the concentration of Mn in the r3-Liq was 0.1 mg/L with a removal efficiency of 73%. Concerning the major cations such as Fe, Mg, and Al, their removal efficiency reached, respectively, 67.76%, 70.01%, and 74.35%, as simplified in Figure 5.

These results indicate that the two-step DP proposed in this study operates as a non-selective decontamination process, allowing the simultaneous removal of trivalent, bivalent, as well as monovalent cations. These results indicate that the precipitation, each time, of phosphorous and calcium ions minimized the content of the hazardous elements retained in the HAp structure.

**Figure 6:**
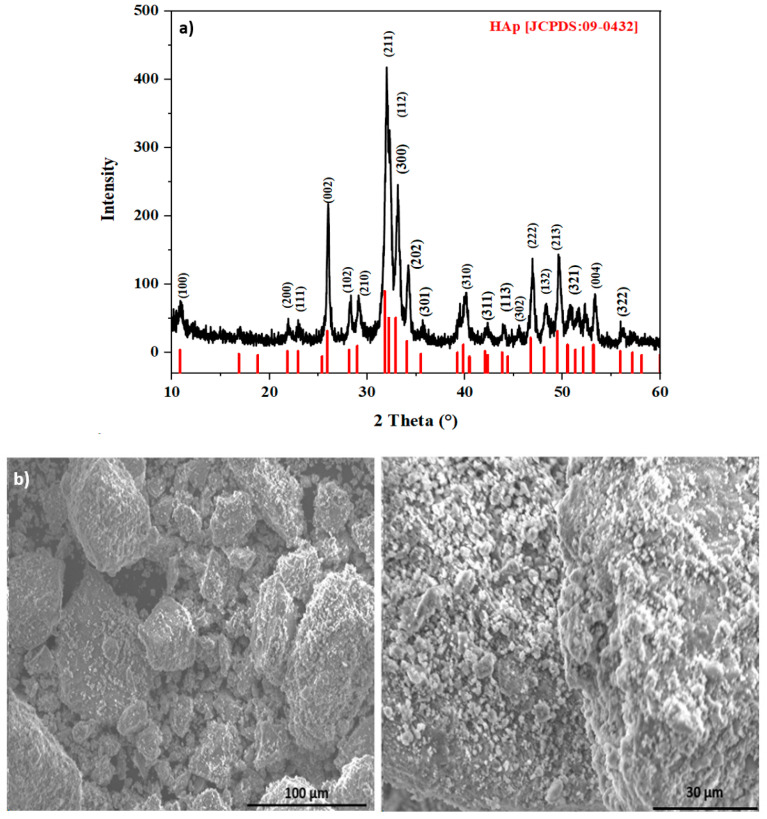
(**a**) XRD pattern and (**b**) SEM images of the precipitated HAp after the third DP cycle.

The XRD pattern (Figure 6) of the solid phase obtained after three consecutive PD cycles exhibited peaks characteristic of a poorly crystalline HAp similar to the first one precipitated from the c-Liq. This suggests that regardless of the number of PD cycles performed, the structure of HAp remains unchanged.

Furthermore, the broad pattern reveals that the precipitated HAp powder has a low crystallinity with nanometric size (33 ± 2 nm) as estimated by the Scherrer formula.

Consequently, HAp, with these attractive properties, is frequently recognized for its high specific surface area [11,42].

Therefore, it can be recommended as a safe and environmentally friendly adsorbent to remove heavy metals and organic pollutants from contaminated aqueous solutions [9]. It can also be proposed as a fertilizer to supply the necessary phosphorus for plant growth [43,44].

The choice of three cycles for the novel purification process was arbitrary, yet the results were promising. These encouraging results indicate that further exploration of this decontamination technique have significant potential to unlock new opportunities in the valorization of natural resources, paving the way for new advancements across various industries.

After the three consecutive PD cycles, PA acid with fewer hazardous elements could be recovered by digesting the purified HAp as another interesting aim of this study. This time, the dissolution step was performed using sulfuric acid instead of nitric acid to convert calcium ions (Ca^2+^) into insoluble calcium sulfate dihydrate (gypsum: CaSO_4_·2H_2_O). The elemental composition of the obtained PA (final liquor: f-Liq) was quantified and is presented in Table 4.

**Figure 7:**
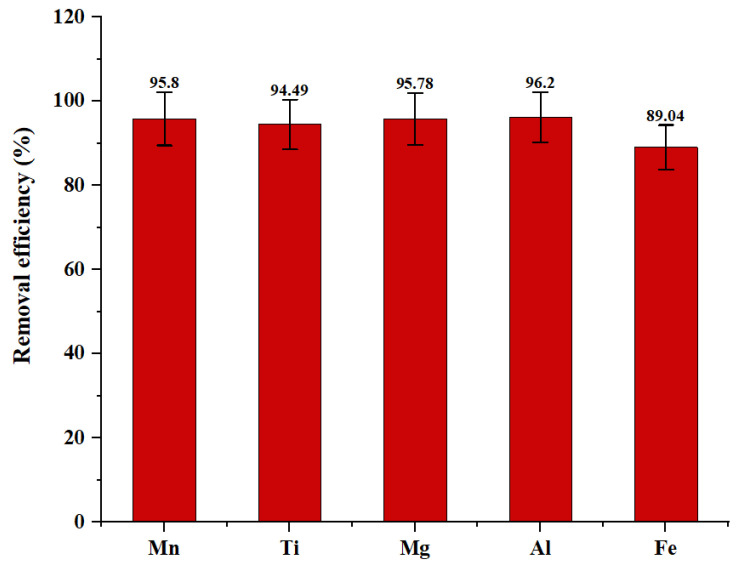
Removal efficiency of different cations in PA.

A remarkable decrease in the concentration of major elements, namely, iron, aluminum, and magnesium, was observed, reaching 0.19, 0.32, and 0.46 mg/L, respectively. Accordingly, the leaching of HAp with sulfuric acid resulted in a removal efficiency of approximately 89% for iron and over 94% for all examined cations, as depicted in Figure 7. It is noted that the precipitation of GP effectively contributed to the absorbance of a significant portion of cations during the leaching process, thereby decreasing their level in the produced phosphoric acid [45,46].

Based on the obtained data, the overall reduction efficiency of hazardous metals in the PA was improved to a very high ratio, and the residual concentration of all elements detected in the f-Liq solution was negligible. It is noteworthy to highlight that the cation content, particularly iron and aluminum, was reduced to the industrially accepted limit of 1.5% [47].

**Figure 8:**
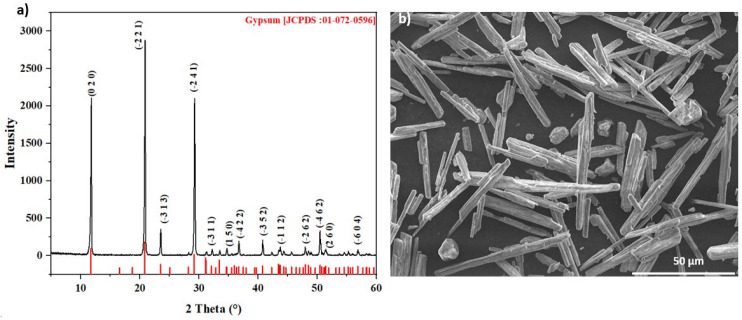
(**a**) XRD pattern and (**b**) SEM image of the obtained GP.

On the other side, GP generated as a byproduct during the digestion of the purified HAp with sulfuric acid was also washed with distilled water and then characterized. The XRD diffractogram provided in Figure 8a indicates the formation of a highly crystalline phase with narrow peaks attributed all to calcium sulfate dihydrate or GP (PDF N°: 01-072-0596). Furthermore, the main mineral impurities usually observed such as brushite (CaHPO_4_·2H_2_O), silicaluminate, or fluorine compounds (CaSiF_6_ or CaF_2_) were absent. The SEM image displayed that needle-shaped crystals having a size varying from 40 to 120 μm (Figure 8b) were obtained.

The chemical composition of the GP as determined by XRF analysis is given in Table 5.

In the light of these results, it is clear that GP is dominated by SO_3_ (44.23%) and CaO (32.63%) as major constituents accompanied with minor impurities including SiO_2_ (0.129%), Al_2_O_3_ (0.01%), P_2_O_5_ (0.016), and Fe_2_O_3_ (0.09). Moreover, the content of Mn and Ti were below the detection limits, confirming their complete elimination.

Based on these results, the generated GP, previously regarded as a waste from the wet process, is now a promising compound following the DP cycles and represents a valuable byproduct of phosphoric acid production with potential applications across various industries. In fact, GP is largely utilized to produce β or α hemihydrate gypsum plaster as a green and low-carbon material in the building sector [48]. Additionally, it can substitute natural GP in the ordinary Portland cement or the manufacturing of porous sound absorbing material [49], supersulfated cement [50], and calcium sulfoaluminate cement [51]. Recently, purified GP attracted significant interest for CO_2_ mineral sequestration due to the important calcium oxide CaO content in GP (around 30 wt.%) that can be applied as raw material for CO_2_ storage [52,53].

Overall, the strategy adopted in this work holds significant promise to solve many environmental challenges and overcome limitations faced by current industrial practices while producing high value-added products through simple and cost-effective protocols. Particularly, with these techniques, we have the flexibility to prepare less-contaminated HAp, offering opportunities for numerous sectors such as agriculture, where it can serve as a fertilizer, environmental applications as an adsorbent, or even in medicine as a biomaterial for bone tissue engineering. Furthermore, digesting purified HAp with sulfuric acid provides a two-fold benefit: generating high-grade PA and GP.

## 3. Materials and Methods

The chemical reagents used in the present work such as nitric acid (HNO_3_, Sigma Aldrich, Taufkirchen, Germany), sulfuric acid (H_2_SO_4_, Sigma Aldrich), and ammonia solution (NH_4_OH, Sigma Aldrich) were all of analytical grades and used without any further purification.

The phosphate rocks were collected from Boukraa region, Laayoune Province, Morocco. Firstly, the collected samples were washed with distilled water to remove the impurities and dried at 120 °C for 24 h, then grounded and calcined at 900 °C for 4 h to remove any organic phases.

### 3.1. Hydroxyapatite Precipitation

The calcined natural samples were sieved to the distribution size of 200–400 µm then reacted with concentrated nitric acid. Specifically, 300 g of the natural phosphate was dissolved in 250 mL of HNO_3_ (2 M) and stirred continuously for 6 h. To intensify the wet process, the reaction was performed under heating (70–80 °C), and the resulting mixture was filtered to obtain the c-Liq according to Equation (1) [54]. Next, the c-Liq was diluted to a final volume of 1 L by adding demineralized water.

Ca_10_(PO_4_)_6_F_2_ + 20 HNO_3_ → 6 H_3_PO_4_ + 10 Ca^2+^ + 20 NO_3_ + 2 HF (1)

The preparation of HAp was performed by adjusting the pH of the diluted c-Liq (source of Ca^2+^ and PO_4_^3−^) to values higher than 9 by adding 2 M ammonia solution under continuous stirring at room temperature (Equation (2)).

6 H_3_PO_4_ + 10 Ca^2+^ + 20 OH^−^ → Ca_10_(PO_4_)_6_(OH)_2_ + 18 H_2_O (2)

The precipitate was filtered and washed three times with hot distilled water to remove any further ions as well as impurities physically adsorbed on the surface powder then dried at 100 °C for 12 h.

Next, a heat treatment was conducted on a fraction of the dried powder at 900 °C for 2 h to better illustrate the crystalline structure of the produced compound.

**Scheme 1:**
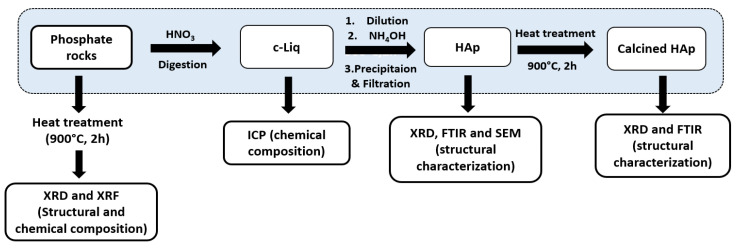
Process schematic for HAp preparation from natural phosphate.

Scheme 1 depicts the sequential steps involved in the HAp precipitation from natural phosphate, along with the characterization techniques applied at each phase.

### 3.2. Purification Process of Hydroxyaptite

**Scheme 2:**
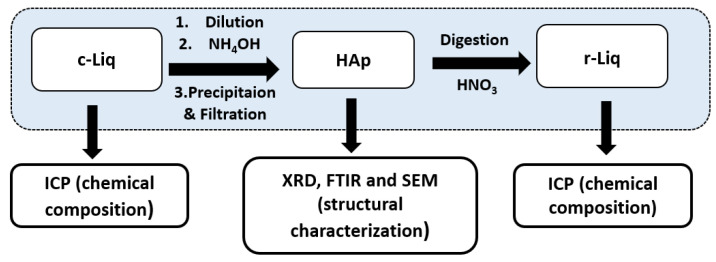
Precipitation and dissolution cycle.

The decontamination procedure adopted herein encompasses two key stages: initially, HAp is precipitated from the c-Liq by treatment with an alkaline medium. Following filtration and washing, the HAp is dissolved in diluted nitric acid at room temperature to produce the r-Liq (Scheme 2).

The chemical composition of the c-Liq and r-Liq was determined, and the removal efficiency of the studied cations was calculated as:

R (%) = ((C_0_ − C_1_)/C_0_) × 100 (3)

where C_0_ and C_1_ are, respectively, the initial and residual concentrations of cations (mg/L).

**Scheme 3:**
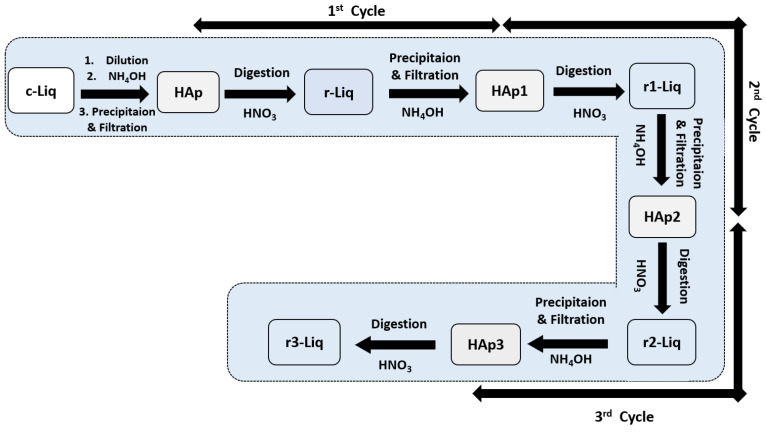
DP cycles for HAp purification.

To intensify the elimination and co-separation of cationic impurities and produce cleaner HAp, series of DP cycles were performed on the same HAp powder at room temperature, as outlined in Scheme 3. The elemental composition of the r-Liq was analyzed for the first and third DP cycles.

### 3.3. Preparation of High-Quality PA and GP

After reducing impurities, the purified HAp (HAp3) was digested with sulfuric acid to generate high-quality PA and GP (Equation (4)) [54]:

Ca_10_(PO_4_)_6_(OH)_2_ + 10 H_2_SO_4_ + 20 H_2_O → 6 H_3_PO_4_ + 10(CaSO_4_, 2H_2_O) + 2H_2_O (4)

**Scheme 4:**
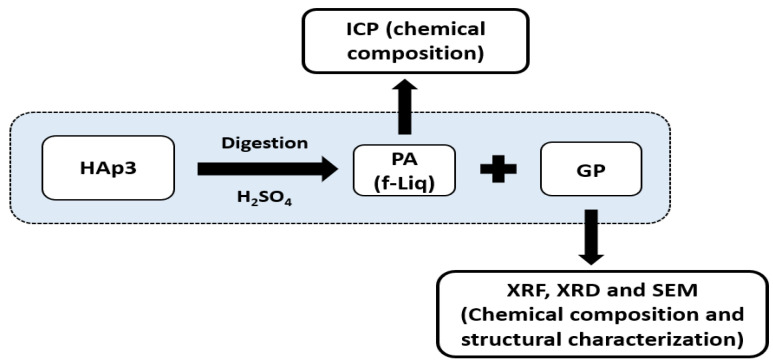
Protocol of the of PA and GP preparation.

By improving the quality of HAp derived from natural phosphate rocks, this process facilitates the production of PA and GP with reduced hazardous elements (Scheme 4).

### 3.4. Characterizations

Chemical analyses of phosphate rocks and produced GP were carried out using X-ray fluorescence spectroscopy (XRF, PANalytical Axios, Eindhoven, The Netherlands). The elemental composition of the produced c-Liq, r-Liq, and PA was performed by Inductively Coupled Plasma Optical Emission Spectrometer (ICP-OES, Horiba Ultima-Expert, Oberursel, Germany). The samples were analyzed to quantify the content of the cations, especially heavy metals (Cd, Pb, Sn, Fe, Al, Ti, Mg, and Mn).

The phase composition of the precipitated and calcined HAp as well as GP was characterized by X-ray Diffraction (Shimadzu XRD 6000, Kyoto, Japan) using monochromatic CuKα radiation (λ = 1.54056 Å) at a voltage of 40 kV and current of 30 mA. All diffractograms were taken in the 2θ range of 10–60° with a step size of 0.02°.

Scherrer formula was applied to estimate the crystallite size of the precipitated HAp particles as follows [42]:

(5) $$D=\frac{0.94\lambda }{\beta (hkl)cos\;\theta }$$

where D is the average crystal size in nm, λ is the wavelength (λ = 1.54056 Å for CuKα radiation), and β(hkl) and θ (in degree) represent, respectively, the full width at half maximum FWHM of the (hkl) reflection and the Bragg angle of the related plan.

Functional groups of the studied samples were identified by Attenuated Total Reflectance Fourier-Transform Infrared (ATR-FTIR) spectroscopy using JASCO Jasco4700-ATR spectrophotometer (Shimadzu, Kyoto, Japan). The spectrum was obtained in the spectral range of 450 to 4000 cm^−1^ at a resolution of 4 cm^−1^ and number of scanning of 32.

Morphological analysis of precipitated HAp and GP was conducted by field emission scanning electron microscopy (FESEM, Quanta 400F, FEI, Eindhoven, The Netherlands).

## 4. Conclusions

This study successfully demonstrated the effectiveness of the precipitation–dissolution technique in reducing the most hazardous cations in HAp derived from natural Moroccan phosphate, paving the way for its potential use in various environmentally friendly applications. The obtained data showed the non-selective nature of the DP process allowing for the elimination of a wide range of cations, reaching removal rates from 100% for Cd, Pb, and Sn to about 70% for Mg, Al, and Fe.

Then, leaching the produced HAp by sulfuric acid enhanced the elimination of the remaining cations to a very high ratio (~95%) while producing high-quality phosphoric acid and gypsum. Therefore, the combination of low cost, high effectiveness, and flexibility makes the dissolution–precipitation strategy a promising approach in the valorization of natural resources for producing attractive products and contributing to environmental protection.

## Figures and Tables

**Table 1:** Chemical composition of calcined phosphate rocks.

Compound	CaO	P_2_O_5_	SiO_2_	F	Al_2_O_3_	MnO	MgO	Fe_2_O_3_	Na_2_O	SO_3_	TiO_2_	Other *
**Concentration (wt%)**	44.01	39.58	5.26	5.12	1.50	0.56	0.40	0.30	0.22	0.64	0.06	0.17

* Other elements include U, K, Sr, Cd, Sn, V, Zn, Cl, and Ni.

**Table 2:** Elemental analysis of the c-Liq performed by ICP-OES.

Elements	Ca	P	Al	Fe	Mg	Mn	Ti	Cd	Sn	Pb
**c-Liq (mg/** **L** **)**	142.4	101	22.983	17.134	14.438	0.369	0.5133	0.1954	0.1	0.05

**Table 3:** Elemental analysis of c-Liq and r-Liq.

Solution	Elements (mg/L)							
	Sn	Pb	Cd	Mn	Ti	Mg	Al	Fe
**c-Liq**	0.1	0.05	0.1954	0.369	0.3234	14.438	22.983	17.134
**r-Liq**	0.053	0.0001	0.0209	0.1315	0.1311	5.416	7.399	7.962
**r1-Liq**		-	0.0023	0.1151	0.09	4.771	6.138	6.513
**r3-Liq**	-	-	-	0.1012	0.007	4.642	5.294	5.013

**Table 4:** Elemental composition of PA.

Solution	Elements (mg/L)				
	Mg	Al	Fe	Mn	Ti
**r3-Liq**	4.242	5.894	5.013	0.1012	0.007
**PA**	0.4647	0.3242	0.1897	0.0009	-

**Table 5:** Chemical composition analysis of GP.

Compound	SO_3_	CaO	Al_2_O_3_	P_2_O_5_	Fe_2_O_3_	Other	LOI ^a^
**Concentration (wt.%)**	48.70	29.74	0.096	0.01602	0.09	0.129	21.23

^a^ LOI: Loss on Ignition.

## Data Availability

Dataset is available upon request from the authors.
